# Diabetes Mellitus is Associated with More Severe Brain Spontaneous Activity Impairment and Gray Matter Loss in Patients with Cirrhosis

**DOI:** 10.1038/s41598-017-08075-x

**Published:** 2017-08-10

**Authors:** Yun Fei Wang, Xiang Kong, Guang Ming Lu, Long Jiang Zhang

**Affiliations:** Department of Medical Imaging, Jinling Hospital, Medical School of Nanjing University, Nanjing, 210002 China

## Abstract

Recent studies showed many cirrhosis patients may have diabetes mellitus (DM), however, the effect of DM on brain in cirrhotic patients is unclear. This study included 34 cirrhosis patients (17 with DM, 17 without DM) and 17 age-, sex-matched healthy controls. MRI examination and neuropsychological tests were performed. Fractional amplitude of low-frequency fluctuation (fALFF) and voxel-based morphometry algorithms were used to obtain fALFF values and gray matter volume, which were compared and correlated with clinical variables. In cirrhosis patients with and without DM, fALFF values were decreased in the left postcentral gyrus, right precentral gyrus, left supramarginal gyrus, bilateral lingual gyri and occipital lobe, while increased in the left orbital frontal gyrus. Gray matter volume was decreased in bilateral caudates and putamen, while increased in bilateral thalami. Compared with non-DM cirrhosis patients, DM cirrhosis patients showed decreased fALFF values in bilateral caudates and decreased gray matter volume in bilateral thalami. The blood glucose levels of cirrhosis patients showed negative correlations with fALFF values in bilateral caudates and gray matter volume in bilateral thalami. In conclusion, DM aggravates brain damage in cirrhosis patients. Thus, it is important to pay more attention to the management of DM in cirrhotic patients.

## Introduction

Liver cirrhosis is a common metabolic disease which leads to multi-system lesions. Brain is one of the affected organs. Numerous studies have reported the adverse effects of liver cirrhosis on structural and functional brain reorganization, thus leading to cognitive dysfunction in these patients^1–4^. Moreover, Garcia *et al*. reported that up to 96% of patients with cirrhosis may be glucose intolerant and 30% patients may have type 2 diabetes mellitus (T2DM)^5^. Another study has demonstrated that coexistent diabetes exacerbates progression of hepatic fibrosis^6^. Recently, a meta-analysis study concluded that T2DM was associated with relative 1.5-fold increased risk for clinically defined Alzheimer disease and 2.5 for vascular dementia compared to non-diabetic individuals^7^. Furthermore, one recent prospective study showed that elevated blood glucose in the absence of DM increased the risk of dementia^8^. Given the high prevalence of DM in the liver cirrhosis population, a better understanding of the impact of DM offers significant opportunities to improve patient’s outcome.

Multimodality MR imaging has been the most important neuroimaging tool to detect the structural and functional changes of human brain in various metabolic brain diseases^9^. For example, voxel-based morphometry (VBM) algorithm is a useful method for evaluating cerebral structural changes in various neurodegenerative diseases^10^, which allows an unbiased search of structural abnormalities across the whole brain^11^. Previous VBM studies showed that loss of brain tissue volume is common in DM and liver cirrhosis^12, 13^. On the other side, resting-state functional MRI (rs-fMRI) allows measuring the spontaneous fluctuations of blood oxygenation level-dependent signal, describing the temporal correlation of neuronal activity across distinct regions^14^. Fractional amplitude of low-frequency fluctuation (fALFF) algorithm is a commonly used method which can provide the information regarding the differences in baseline brain activity involved in physiological and pathological states^15^. It has been demonstrated that brain resting state functional connectivity coupled with structural connectivity^16^. This coupling was disrupted in various brain diseases^17–20^.

Because both brain atrophy and cognitive impairments occur in patients with cirrhosis and DM, thus, in the current study, we have a hypothesis that the presence of T2MD may increase the risk of complication in the brain of cirrhosis patients who may suffer from much more severe brain structural and functional abnormality. To our knowledge, no neuroimaging studies have been published to study the effects of DM on brain structural and functional reorganization in the patients with liver cirrhosis. In this study, we combined VBM and fALFF algorithms to simultaneously quantify gray matter volume and spontaneous brain activity damage to define the effect of DM on brain structural and functional changes in cirrhosis patients.

## Results

### Demographics, Clinical and Neuropsychological Data

Demographic, clinical and neuropsychological data of the three groups are listed in Table 1. The differences were not found for age or gender among the three groups (both P > 0.05). Both patient groups had a poorer performance for NCT-A and DST than the controls (both P < 0.05). However, for the comparison between the patient groups, diabetic cirrhosis patients showed the worse performance in the neuropsychological tests than non- diabetic cirrhosis patients (both P < 0.05).

**Table 1:** Demographic, clinical and neuropsychological data of the three groups

Variables	Diabetic cirrhosis (n = 17)	Non-diabetic cirrhosis (n = 17)	Healthy controls (n = 17)	P *valu*e
Age (y)	54.8 ± 8.3	53.4 ± 5.9	54.4 ± 7.9	0.842^a^
Gender (M/F)	12/5	12/5	12/5	0.999^b^
NCT-A (s)	52.8 ± 20.0	48.1 ± 12.8	36.8 ± 9.1	0.018^c^
DST (n)	34.5 ± 10.9	40.5 ± 12.5	52.4 ± 8.9	0.001^a^
Child-Pugh scale (A/B/C)	15/1/1	15/1/1	none	0.999^c^
HE (N/M/S)	15/1/1	15/1/1	none	0.999^c^
Ascites (N/M/S)	9/5/3	11/6/0	none	0.193^c^
TB (μ mol/L)	26.9 (36.2−15.8)	28.2 (38−17.1)	none	0.692^c^
Albumin (g/L)	35.8 ± 6.5	35.1 ± 6.0	none	0.804^d^
PT (s)	14.1 (15.5−13.1)	16.2 ± 3.0	none	0.063^c^
Ammonia (μ mol/L)	54.5 ± 24.7	39 (76.5−11.5)	none	0.480^c^
ALT (U/L)	39.6 ± 27.9	31 (39−22)	none	0.823^c^
AST (U/L)	41.6 ± 21.3	39 (55−27.5)	none	0.796^c^
Glucose (mmol/L)	7.3 ± 1.3	4.6 (5.1−4.2)	none	0.001^c^
Cirrhosis duration (y)	3.2 ± 2.9	2.4 ± 2.5	none	0.380^d^

Mean ± standard deviation; Median and inter-quartile range [M (QU-QL)].

P values indicate significant differences across groups.

^a^analysis of variance; ^b^chi square test; ^c^k-independent samples nonparametric tests ^d^two-sample t tests.

y = year; M = male; F = female; NCT-A = number connection test type A; DST = digit symbol test; s = second; n = number; HE = hepatic encephalopathy; N = none; M = mild; S = severe; TB = total bilirubin; PT = prothrombin time; ALT = alanine transaminase; AST = aspartate transaminase.

### Regional gray matter volume differences of the three groups

**Figure 1:**
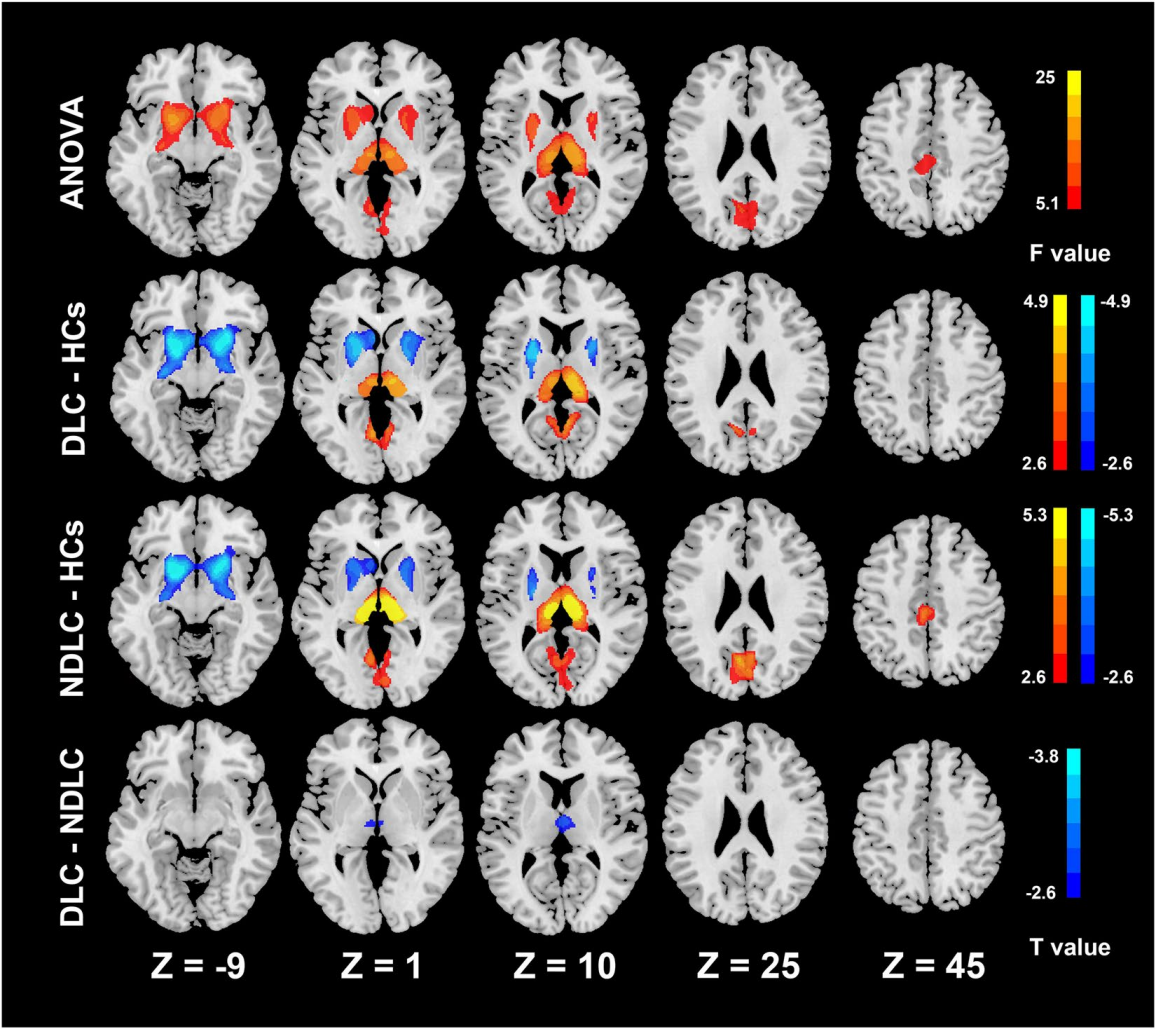
Group differences of gray matter volume. ANOVA analysis shows the volume of the putamen, thalamus, caudate, cuneus, calcarine, middle cingulate cortex and bilateral lingual gyri have significant differences among three groups. Compared with healthy controls, the diabetic cirrhosis patients group shows decreased volume in the caudate and putamen and increased volume in the thalamus and lingual gyrus. Non-diabetic cirrhosis patients group has decreased volume in the caudate and putamen and increased volume in the thalamus, middle cingulate cortex, cuneus and calcarines. The diabetic cirrhosis patients group has decreased volume in the thalamus compared with the non-diabetic cirrhosis patients group (all p < 0.05). ANOVA = analysis of variance, DLC = diabetic liver cirrhosis, NDLC = non-diabetic liver cirrhosis, HCs = healthy controls.

Compared with healthy controls, diabetic cirrhosis patients exhibited diffuse bilaterally symmetrical decreased gray matter (labeled in cold color) volume in the bilateral caudates, putamen and lingual gyri and increased volume (labeled in warm color) in bilateral thalami. Non-diabetic cirrhosis patients exhibited similar gray matter volume changes (Table 2). In the comparison between the two patient groups, diabetic cirrhosis patients presented bilaterally decreased gray matter volume of thalamus compared with non-diabetic cirrhosis patients (Fig. 1, Table 2).

**Table 2:** Differences of VBM among three groups.

Anatomic Regions	Diabetic cirrhosis vs healthy controls	Non-diabetic cirrhosis vs healthy controls	Diabetic vs Non-diabetic
	MNI Coordinates (x, y, z)/t Value	MNI Coordinates (x, y, z)/t Value	MNI Coordinates (x, y, z)/t Value
Cingulum_Mid	—	(−4,−28,46)/3.72	—
Cuneus	—	(−5,−65,24)/4.56	—
Calcarines	—	(−3,−65,12)/4.01	—
Caudate_L	(−5,16,2)/−3.92	(−7,11,2)/−3.21	—
Caudate_R	(8,16,−2)/−3.35	(13,16,−4)/−3.28	—
Thalamus_L	(−6,−19,13)/3.95	(−11,−28,10)/5.29	(−6,−15,2)/−2.73
Thalamus_R	(8,−16,18)/4.93	(11,−22,10)/5.29	(7,−14,12)/−2.87
Putamen_L	(−18,6,−7)/−5.04	(−19,8,−9)/−5.29	—
Putamen_R	(19,12,−7)/−4.74	(18,12,−9)/−5.04	—
Lingual_L	(−6,−64,4)/3.65	—	—
Lingual_R	(6,−62,4)/3.89	—	—

All P < 0.05, with Alphasim corrected.

MNI = Montreal Neurological Institute; Mid = middle;L = left; R = right.

**Table 3:** Differences of fALFF among three groups.

Anatomic Regions	Diabetic cirrhosis vs healthy controls	Non-diabetic cirrhosis vs healthy controls	Diabetic vs Non-diabetic
	MNI Coordinates (x, y, z)/t Value	MNI Coordinates (x, y, z)/t Value	MNI Coordinates (x, y, z)/t Value
Postcentral_L	(−46,−23,44)/−3.98	(−48,−23,43)/−3.77	—
Precentral_R	(48,−12,48)/−3.65	(38,−14,55)/−3.62	—
SupraMarginal_L	(−54,−21,36)/−4.34	(−53,−20,36)/−3.61	—
Cuneus	(2,−87,22)/−3.72	—	—
Calcarines	(1,−89,−2)/−3.61	—	—
Caudate_L	—	(−10,12,0)/3.12	(−9,9,0)/−3.81
Caudate_R	—	(12,12,−9)/3.50	(8,12,2)/−3.48
Occipital_Mid_L	(−23,−95,−2)/−4.27	(−40,−73,2)/−3.80	—
Occipital_Mid_R	(43,−82,4)/−3.48	(41,−82,5)/−2.97	—
Occipital_Inf_L	(−27,−95,−7)/−3.90	(−24,−93,4)/−2.88	—
Occipital_Inf_R	(30,−9,−12)/−4.25	(26,−96,−10)/−3.68	—
Lingual_L	(−29,−92,−12)/−4.14	(−32,−91,−14)/−3.52	—
Lingual_R	(28,−94,−14)/−3.97	(24,−96,−12)/−3.82	—
Frontal_Sup_Orb_L	(−12,18,−21)/3.92	(−16,20,−18)/2.96	—

All P < 0.05, with Alphasim corrected.

MNI = Montreal Neurological Institute; L = left; R = right; Mid = middle; Inf = inferior; Sup = superior; Orb = orbital.

### fALFF differences of the three groups

**Figure 2:**
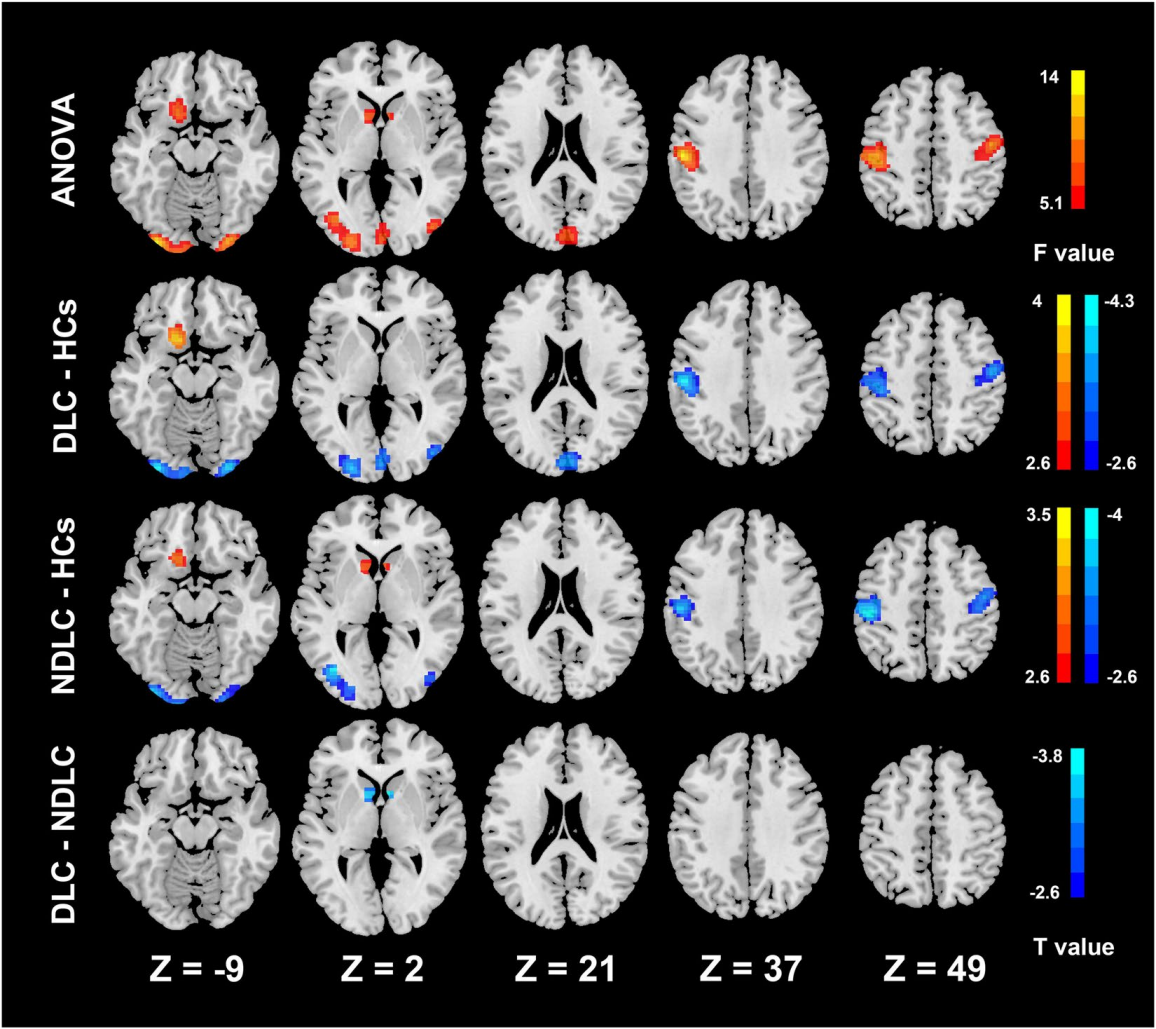
Group differences of fALFF. ANOVA analysis shows fALFF values of the caudate, occipital lobe, left postcentral gyrus, right precentral gyrus, left supramarginal gyrus and left frontal lobe have significant differences among three groups. Compared with controls, the diabetic cirrhosis patients group shows decreased fALFF values in the occipital lobe, left postcentral gyrus, right precentral gyrus and left supramarginal gyrus and increased fALFF values in the frontal lobe. Non-diabetic cirrhosis patients group has decreased fALFF values in the occipital lobe, left postcentral gyrus, right precentral gyrus and left supramarginal gyrus and increased fALFF values in the caudate and left frontal lobe. The diabetic cirrhosis patients group has decreased fALFF values in the caudate compared with the non-diabetic cirrhosis patients group (all p < 0.05). ANOVA = analysis of variance, DLC = diabetic liver cirrhosis, NDLC = non-diabetic liver cirrhosis, HCs = healthy controls.

Compared to controls, patients with liver cirrhosis showed widespread fALFF differences (P < 0.05, Alphasim corrected, cluster size > 98). These two patients groups showed both decreased fALFF value (labeled in cold color) in both cortical and subcortical regions (including the left postcentral gyrus, right precentral gyrus, left supramarginal gyrus, occipital lobe, and bilateral lingual gyri) and increased fALFF value (labeled in warm color) in the left frontal lobe. In the comparison between the patient groups, diabetic cirrhosis patients presented decreased fALFF signals in the bilateral caudates compared with non-diabetic cirrhosis patients (Fig. 2, Table 3).

### Correlation analysis results

**Figure 3:**
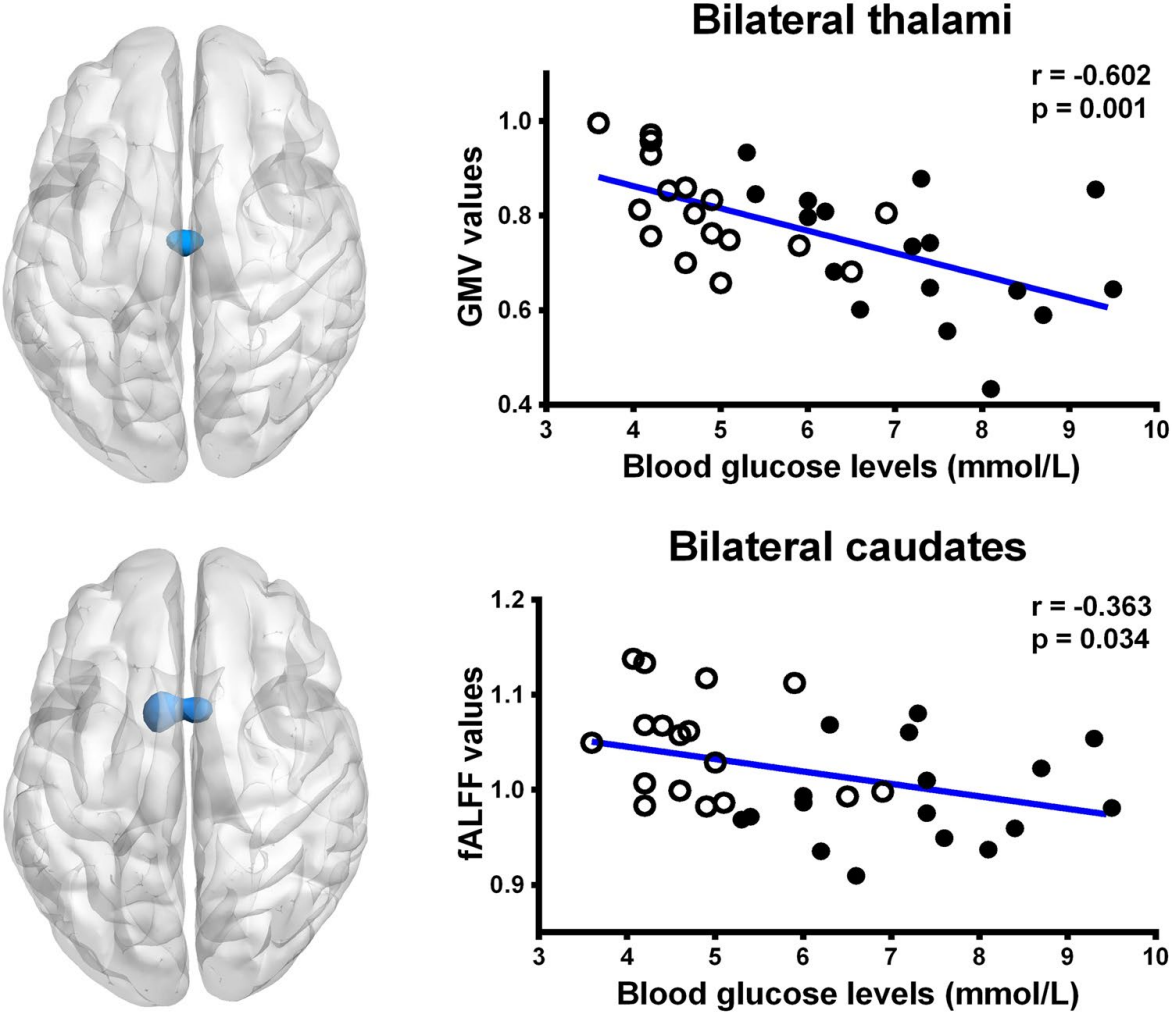
Correlation results in all cirrhosis patients. There was a negative correlation between the blood glucose levels and gray matter volume in bilateral thalami (r = −0.602, P = 0.001) and the fALFF values (r = −0.363, P = 0.034) in the bilateral caudates in all cirrhosis patients. GMV = gray matter volume; fALFF = fractional amplitude of low-frequency fluctuation; ⚪ = diabetic cirrhosis patient; ● = non-diabetic cirrhosis patient.

**Figure 4:**
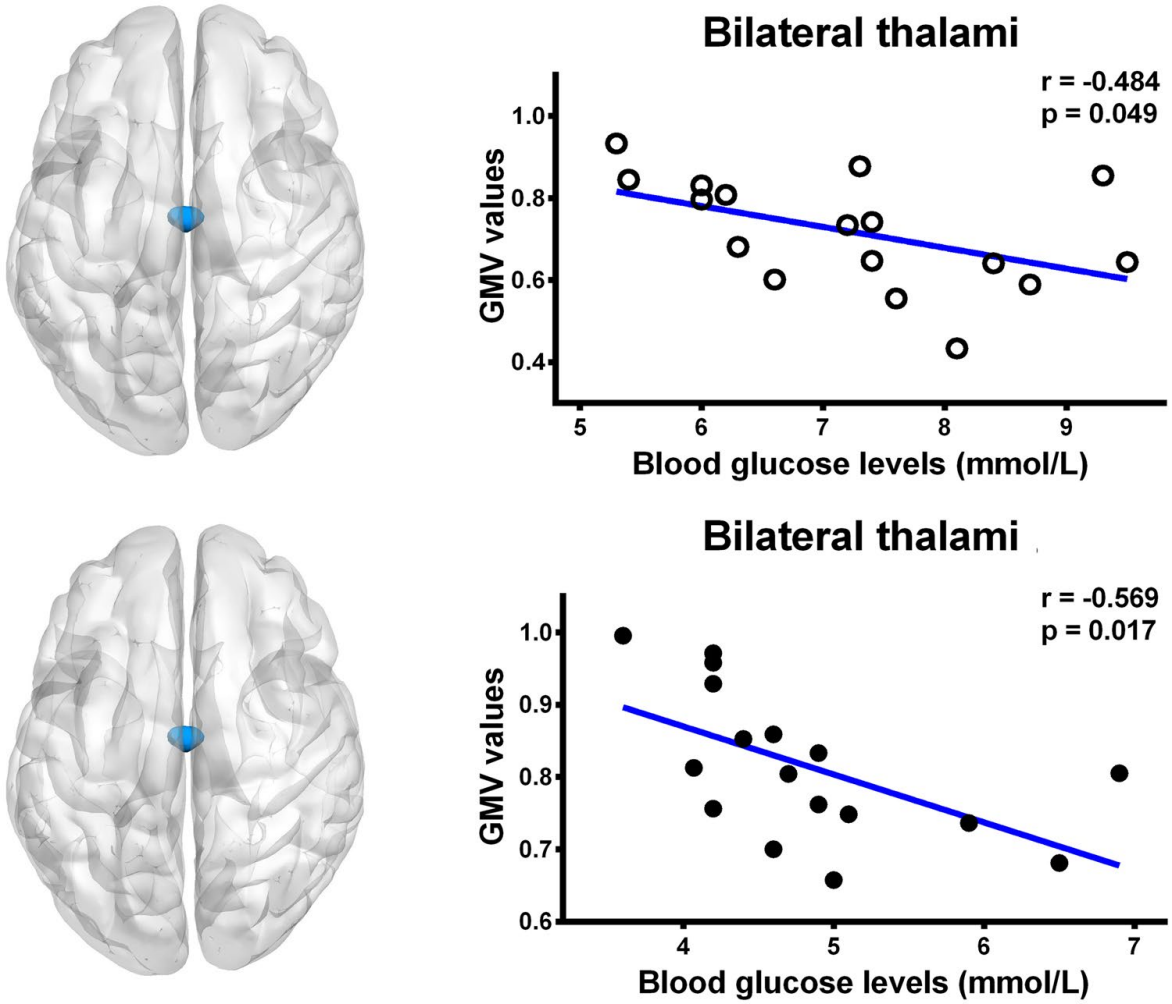
Correlation results in diabetic cirrhosis patients and non-diabetic cirrhosis patients. There was a negative correlation between the blood glucose levels and gray matter volume in bilateral thalami in diabetic cirrhosis patients group (r = −0.484, P = 0.049) and in non-diabetic cirrhosis patients group (r = −0.569, P = 0.017). GMV = gray matter volume; ⚪ = diabetic cirrhosis patient; ● = non-diabetic cirrhosis patient.

There was a negative correlation between the blood glucose levels and the fALFF values in the bilateral caudates in all cirrhosis patients (r = −0.363, P = 0.034) (Fig. 3). The cirrhosis patients group also had a negative correlation between the blood glucose levels and the volume of the bilateral thalami (r = −0.602, P = 0.001) (Fig. 3). However, when correlating the blood glucose levels within each patient group, only bilateral thalamus volume showed negative correlation in diabetic cirrhosis patients group (r = −0.484, P = 0.049) and non-diabetic cirrhosis patients group (r = −0.569, P = 0.017) (Fig. 4). No relationships were identified in the cirrhosis patients between neuropsychological test scores, venous blood ammonia level and gray matter volume, or fALFF values in other regions which showed the statistical differences (all P > 0.05).

## Discussion

In the present study, we found widespread gray matter volume changes and abnormal fALFF signals, which were much more severe in diabetic cirrhosis patients. Moreover, we also found that the blood glucose levels were correlated with brain gray matter volume and spontaneous neuronal activity changes in cirrhotic patients.

Our findings show that liver cirrhosis is associated with significantly greater global gray matter loss, especially in the basal ganglia. Guevara *et al*.^21^ found the loss of brain tissue volume was common in liver cirrhosis patients, which mainly in the frontal and parietal regions and putamen for gray matter, and their results were similar as our findings. Other studies also reported decreased gray matter volume in the globus pallidus, putamen and caudate^1, 3, 22^. Persistent abnormal metabolism changes, manifested as elevated deposition of manganese^23, 24^ may lead to permanent cell damage in the basal ganglia^25, 26^. Another VBM study^13^ demonstrated that the decreased gray matter volume was related to persistent exposure to hyperammonaemia, which indicated that ammonia-related cerebral edema might lead to gray matter atrophy. However, we did not find a significant correlation between venous blood ammonia level and decreased gray matter volume in our study. Difference of inclusion criteria for patient cohorts between these two studies may account for the inconsistent findings.

We found increased thalamus volume in cirrhotic patients compared with controls, which is also in line with previous findings^27^. According to our current knowledge, the thalamus serves as a major relay station in the brain which integrates signal input from numerous cortical regions and facilitates their communications^28^. One plausible interpretation is that increased thalamus volume, accompanied by decreased bilateral putamen and caudate volumes, is a reactive hypertrophy of relay nuclei within cortical-basal ganglia–thalamic circuits^29, 30^. Another possible reason for the thalamic enlargement is the compensatory effect for the basal ganglia dysfunction, which is a common finding (symmetric basal ganglia hyperintensity in T1 weighted images) in cirrhosis patients^31^. Further subgroup analysis showed decreased volume of the thalamus in diabetic cirrhosis patients compared with non-diabetic cirrhosis patients. This is a very interesting finding in our study. Decreased thalamus volume in diabetic cirrhosis patients can be resulted from insulin resistance in T2DM which could reduce glucose metabolism in the brain tissues^32^, thus inhibiting the compensatory mechanisms of the thalamus. As a consequence, the increased thalamus volumes were decreased in diabetic cirrhosis patients compared with non-diabetic cirrhosis patients. Thalamus atrophy has been reported in type 1 diabetes in a recent meta analysis^33^. However, further studies with large sample size are needed to uncover this interesting finding.

Amplitude of low-frequency fluctuation (ALFF) algorithm proposed by Zang *et al*.^34^ has been widely used in functional MR imaging studies. However, ALFF has some limitations in itself, such as sensitive to the physiological noise. Thus, Zou *et al*. proposed the fALFF algorithm to improve these shortcomings of ALFF^15^. The fALFF algorithm is an advanced technique which can effectively minimize the physiological related noise around the major vessels, thus providing a more specific index of low frequency oscillatory phenomena. In contrast to the traditional measurement of the ALFF, fALFF measurements are more reliable and more specific to reflect intrinsic neural activities^15, 35^. In this study, we found that both patient groups showed the similar patterns of fALFF alterations. The main difference between two patient groups was that the diabetic cirrhosis patients showed decreased fALFF signals in the bilateral caudates when compared with non-diabetic cirrhosis patients. We speculate that the decreased fALFF in the occipital regions may underline the potential cognitive abnormalities relative to visual function which was reflected by the NCT-A and DST. And the increased fALFF signals in the frontal lobe may be a compensatory effect for the visual function decline, which represents cortical plasticity and reorganization in cirrhosis patients. However, in the comparison between the patient groups, diabetic cirrhosis patients showed decreased fALFF signals in the subcortical regions (bilateral caudates). It is possible that the basal ganglia are broadly responsible for sensorimotor coordination and the caudate nucleus is more engaged in goal-directed action^36^. In this study, the decreased activation in caudate nucleus may reflect the loss or decreased energy of neurons, which may be responsible for the worse performance in neuropsychological tests in diabetic cirrhosis patient group. Additionally, we found gray matter volume loss in the bilateral thalami, however, no significant fALFF changes were observed when compared with the controls in the same brain regions.

The most structural and functional changes are distributed in different brain areas in our study. However, we also found some brain areas showed both fALFF and gray matter volume changes, including the bilateral lingual gyri in the diabetic cirrhosis patients group and the bilateral caudates in the non-diabetic cirrhosis patients group. Although some studies have reported that functional connectivity coupled with structural connectivity^16, 18, 19^, we did not find significant correlation between the fALFF changes and gray matter volume changes in these areas. We speculate that the two methods used in our study did not reflect the connectivity changes in the brain, which may account for the different abnormal brain areas involved in the structural and functional changes.

Several limitations must be considered in our study. First, the sample size of our study is relatively small, which may limit the statistical power in the subgroup analysis. It is needed to further collect larger cohort to solve this issue in future. Second, our study was consisted of a heterogeneous patient cohort, with 4 patients having a history of hepatic encephalopathy among the cirrhotic patients. Although we strictly matched this variable, the wide spectrum of disease state may produce the bias in our results. Third, we used only two neuropsychological tests to assess cognitive function of the patients; although they were widely used in studies regarding liver cirrhosis and recommended by the working party of 11^th^ world congress of gastroenterology. These two tests can not detect all the cognitive impairments induced by DM and cirrhosis. Thus, more neuropsychological tests are acquired to comprehensively evaluate the cognitive status of cirrhosis patients with DM in the future. Lastly, lactulose or other medical treatments were administrated in almost all patients, which can affect rain structural and functional changes in cirrhotic patients. However, it is rather difficult to avoid these factors because the measurements have to be administrated to prevent the deterioration of diseases.

In summary, by applying both VBM and fALFF algorithms, we found brain functional and structural abnormalities were apparent and bilaterally symmetrical in cirrhotic patients, which were more widespread in cirrhosis patients with DM than those without DM. The blood glucose levels are correlated with the brain structural and functional changes in both patient groups. These findings suggest that more attention is needed in the management of DM in cirrhotic patients.

## Material and Methods

### Participants

This is a retrospective study, which was approved by Ethics Committee of Jinling Hospital. All participants gave written informed consent before MR imaging and all methods were performed in accordance with the relevant guidelines and regulations. This study included 34 subjects with liver cirrhosis, who were subdivided into two groups according to DM history, i.e.,17 cirrhotic patients with DM, 17 cirrhotic patients without DM and 17 age, sex matched healthy controls. DM status was verified according to the following criteria: fasting plasma glucose level higher than 126 mg/dL, a non-fasting plasma glucose level higher than 200 mg/dL, or self-reported administration of anti-diabetes drugs such as insulin therapy. Each group comprised of 12 men and 5 women, who were right-handed. We also strictly matched the prevalence of overt hepatic encephalopathy and Child-Pugh scale in the two patient groups to avoid the effect of these factors on the results of cognitive assessment (Table 1). Cirrhosis was diagnosed by liver biopsy or based on the presence of biochemical, ultrasonographic, CT or MR imaging or endoscopic features of portal hypertension and/or liver dysfunction. Diabetes status was defined by subject self-report during a standardized interview (All 17 diabetic patients had T2DM). The inclusion criteria were as follows: subjects who were 18 years or older, they could finish MR examination without any MR imaging contraindications. Exclusion criteria included: (1) any drug abuse history; (2) head trauma history; (3) neurological or psychiatric disorders; (4) visual or auditory deficits; (5) rotation more than 1.0° or translation more than 1.0 mm during MR scanning.

### Neuropsychological Assessments

Before MR examinations, two neuropsychological tests [number connecting test type A (NCT-A) and digit symbol test (DST)] recommended by the working party of 11^th^ World Congress of Gastroenterology^37^ were assessed according to the standard methods for all subjects.

### Laboratory Examinations

Before MR scanning, blood samples of all patients were taken and blood biochemistry tests were underwent, including albumin, prothrombin time, bilirubin metabolism tests (including total bilirubin, indirect bilirubin, and direct bilirubin), glutamic oxalacetic transaminase ammonia, glutamic pyruvic transaminase, and fasting glucose levels. The severity of liver disease was determined according to Child-Pugh score. According to this scoring system, 15 patients had Child-Pugh grade A, 1 Child-Pugh grade B and 1 grade C in each patient group, respectively. All laboratory tests were unavailable for the controls.

### MR imaging data acquisition

All MR imaging data were collected in a 3.0 Tesla scanner (TIM Trio, Siemens Medical Solutions, Erlangen, Germany), which equipped with the standard 12-channel head coil. First, high resolution T1 weighted structural images were acquired in the sagittal orientation with a magnetization prepared rapid gradient echo sequence (TR/TE = 2300 ms/2.98 ms, field of view (FOV) = 256 mm × 256 mm, flip angle = 9°, slice thickness = 1 mm, voxel of 1.0 mm × 1.0 mm × 1.0 mm, acquisition matrix = 256 × 256, 176 slices). A single-shot, gradient-recalled echo planar imaging sequence was used to acquire functional images (TR/TE = 2000 ms/30 ms, FOV = 240 mm × 240 mm, flip angle = 90°, voxel size = 3.75 mm × 3.75 mm × 4 mm, image matrix = 64 × 64, 30 axial slices) aligned along the anterior-posterior commissure to cover the whole brain. The rs-fMRI scan lasted for 500 seconds and 250 brain volumes were collected. Then, images with axial T2 fluid attenuated inversion recovery sequence (TR/TE = 9000 ms /93 ms, inversion time = 2500 ms, flip angle = 130°, thickness/gap = 4.0 mm /1.2 mm, matrix = 232 × 256, FOV = 220 mm × 220 mm) was conducted to exclude clinically silent lesions. The subjects were told to keep awake, relax with their eyes closed, heads still and no thinking anything else during the functional MRI scans.

### Data processing for VBM analysis

VBM data analysis was performed using the VBM8 toolbox in the Statistical Parametric Mapping (SPM8, http://www.fil.ion.ucl.ac.uk/spm) software. Firstly, structural MR imaging data were spatially normalized into standard Montreal Neurological Institute (MNI) space. Subsequently, MR images were segmented into gray and white matter and cerebrospinal fluid using the new Segment and DARTEL modules included in SPM8. Finally, the resultant images were smoothed with full width at half maximum (FWHM) Gaussian kernel of 8 mm.

### Data processing for fALFF analysis

Image preprocessing for fALFF data was carried out using Data Processing Assistant for Resting State fMRI (DPARSF) and SPM8^38^. For each subject, the beginning ten volumes were discarded to ensure the subjects can adapt to scanning noise. Slice-timing and realignment were then performed in SPM8. Then, the functional images were coregistered to the individual 3D T1 weighted images. The resulting normalized functional data were spatially smoothed with FWHM Gaussian kernels of 6 mm, while linear trends of time courses were removed. Finally, all images underwent temporally filtered (0.01–0.1 Hz) to remove the effects of high-frequency noises and very low-frequency drift.

For fALFF analysis, with a fast Fourier transform, the time series of each voxel was transformed to the frequency domain. Then we calculated the sum of the frequencies in the low frequency band (0.01–0.1 Hz). We also computed the power spectrum and performed square root transform at each voxel. The averaged square root was regarded as ALFF value. For each subject, ALFF value of each voxel was normalized by the mean within-brain ALFF value. The fALFF value is the ratio between the sum of power spectrum of low-frequency (0.01–0.1 Hz) and the sum of the entire frequency range.

### Statistical Analysis

Analysis of demographic, neuropsychological scores and clinical variables was performed with SPSS software (version 18 SPSS, IBM). Gender difference among three groups was evaluated with χ^2^ test. The normality of quantitative data was analyzed with Kolmogorov-Smirnov test. Normally distributed data, expressed as mean ± standard deviation (SD), were analyzed by using ANOVA test. The variance homogeneity of these data was examined with Bartlett test. The Fisher test was used if the variance was homogeneous, or else, Brown-Forsythe approximate variance analysis was used. Non-normal distribution data, reported as median and inter-quartile range [M (QU-QL)], were analyzed with independent sample nonparametric test. If ANOVA test showed the significant differences, we further used post hoc analysis to perform inter-group comparisons. A P value < 0.05 was considered as statistical difference.

We used the dpabi software package to analyze structural and functional imaging data^39^. To assess the effect of comorbid DM on spontaneous neuronal activity and gray matter volume in cirrhosis patients, smoothed images were analyzed first by an ANOVA for the three groups, followed by post hoc unpaired two-sample t-tests. Sex and age were imported as nuisance covariates for all inter-group analyses. All the analysis results were regarded as statistically significant at P values < 0.05 (Alphasim correction) and corrected for multiple comparisons with least significant difference (LSD). LSD is a common post hoc multiple comparison tests, which is recommended by the dpabi software (41) and applied in lots of brain MR studies^40^. Clinical variables and neuropsychological scores (including NCT-A and DST) were correlated with imaging parameters which showed the statistical difference in aforementioned ANOVA results in the patient groups using Pearson correlation analysis.

### Data Availability

The datasets generated during and/or analysed during the current study are available from the corresponding author on reasonable request.
